# Preventing water-inrush from floor in coal working face with paste-like backfill technology

**DOI:** 10.1038/s41598-023-43311-7

**Published:** 2023-09-24

**Authors:** Xingyue Qu, Longqing Shi, Jin Han

**Affiliations:** 1 College of Earth Science and Engineering, Shandong University of Science and Technology, Qingdao, 266590 China; 2 College of Computer Science and Engineering, Shandong University of Science and Technology, Qingdao, 266590 China

**Keywords:** Risk factors, Engineering

## Abstract

In the view of the situation where great economic loss often occurs during mining deep coal seams in Feicheng coal field due to water inrush from the floor Ordovician limestone aquifer, the floor "lower four-zone" theory was used as a guide. 81006 working face of Caozhuang Coalmine in Feicheng coal field was taken as the research background, and paste filling technology was proposed to inhibit or reduce the damaged floor depth caused by mine ground pressure in order to prevent water inrush from the floor Ordovician limestone aquifer. Glue material, coal gangue powder, and fly ash were selected as filling material, and a ground filling system, including a material production system, storage material system, power supply and water supply system, automatic control and measurement system, monitoring and control communications system, emergency response system, and underground filling system, including pipe conveyor system and working face blocking grout loss system, were established to achieve effective filling goaf. Field stress monitoring and floor damaged depth measurement showed that when reaching a steady state after a period of time, paste filling working face not only restored to the original stress state but also significantly reduced the floor's damaged depth caused by mine ground pressure. This verifies that working face with paste-like backfill technology is a very effective measure to prevent water-inrush from the floor.

## Introduction

**Figure 1:**
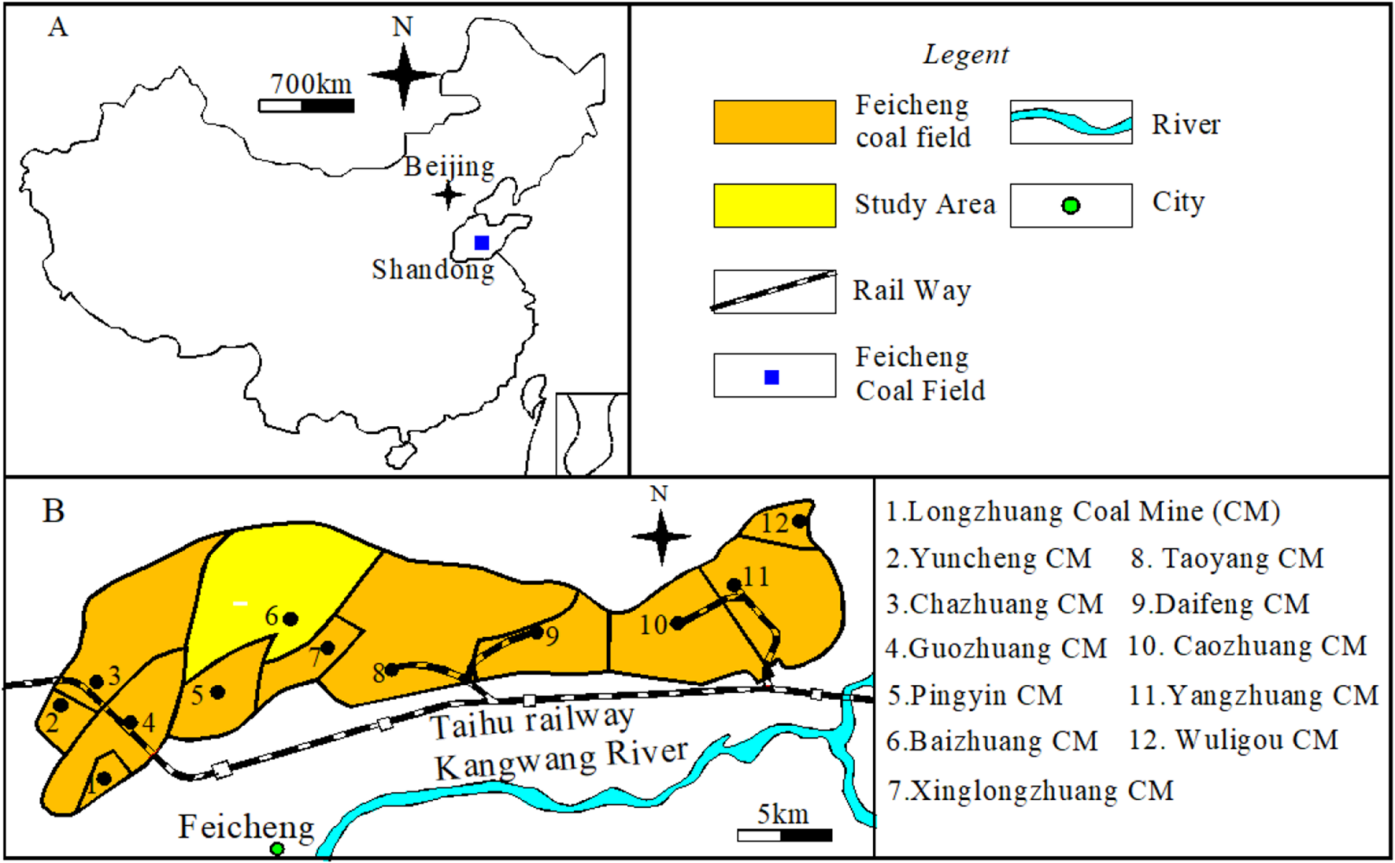
Geographic location of Caozhuang Coalmine in China.

Feicheng coal field, located in Feicheng city, Shandong province, has geographical coordinates of longitude 116°41′05" and latitude 36°12′45", covering an area of 98 km^2^ (Fig. 1A). The mining of deep coal seams in Feicheng coal field is seriously threatened by water inrush from the floor Ordovician limestone aquifer. Approximately 65% of the recoverable coal reserves are in the danger zone of Ordovician limestone aquifer water inrush, with 285 water inrush cases having occurred since 1960, of which 279 cases belong to floor water inrush from Ordovician limestone karst^1–3^. In seven cases, the amount of water inrush from the floor exceeded 1000 m^3^/h and flooded six coal mines^4,5^. The largest amount of water inrush from floor Ordovician limestone aquifer occurred on January 5, 1993, in Guojiazhuang Coal Mine, with a volume of 32,970 m^3^/h, causing a direct economic loss of 11.0179 U.S. dollars^6–9^. Feicheng coal field contains six minable coal seams, namely Permian coal seam #3, Carboniferous coal seam #7, coal seam #8, coal seam #9, and coal seam #10. Coal seams #3 and #7 have already been mined out, coal seams #9 and #10 have not been mined yet, while coal seam #8 is currently being mined. Caozhuang Coalmine, located in the east of Feicheng coal field (Fig. 1B), often experiences mine water inrush from the floor during the mining of coal seam #8. This is mainly due to the deep destroyed floor caused by underground pressure during mining of coal seam #8 in Caozhuang Coalmine, which leads to a decrease in the effective thickness of the water-resisting layer between coal seam #8 and floor No. 5 limestone aquifer and Ordovician limestone aquifer^4^. Based on reference literature^10–23^, we collaborated with Caozhuang Coalmine to study paste-like backfill technology in 81006 coal working face, aiming to reduce the depth of destroyed floor and prevent water inrush from the floor.

### A profile of 81006 working face

**Figure 2:**
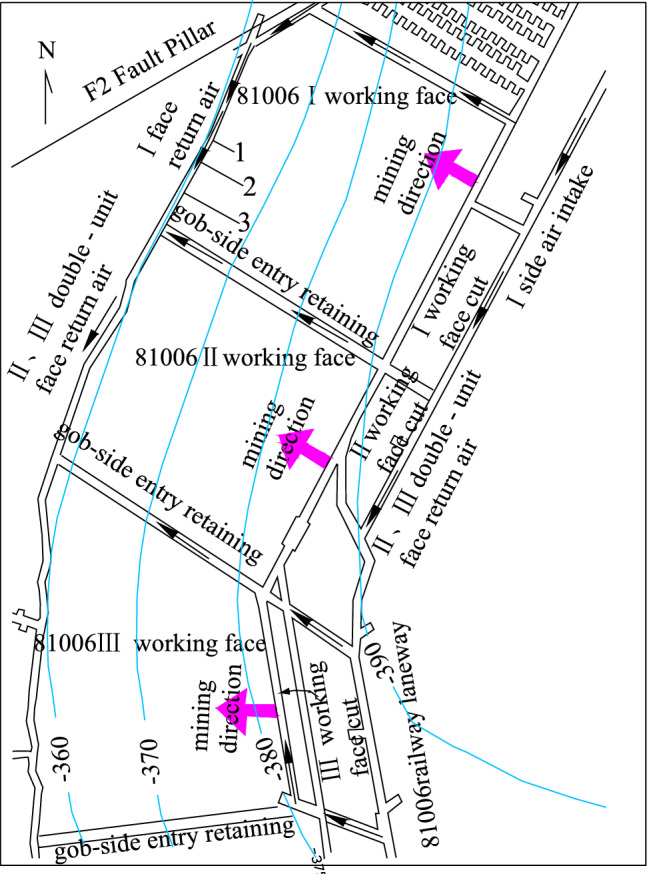
Backfill mining layout plans of 81006 working face.

**Figure 3:**
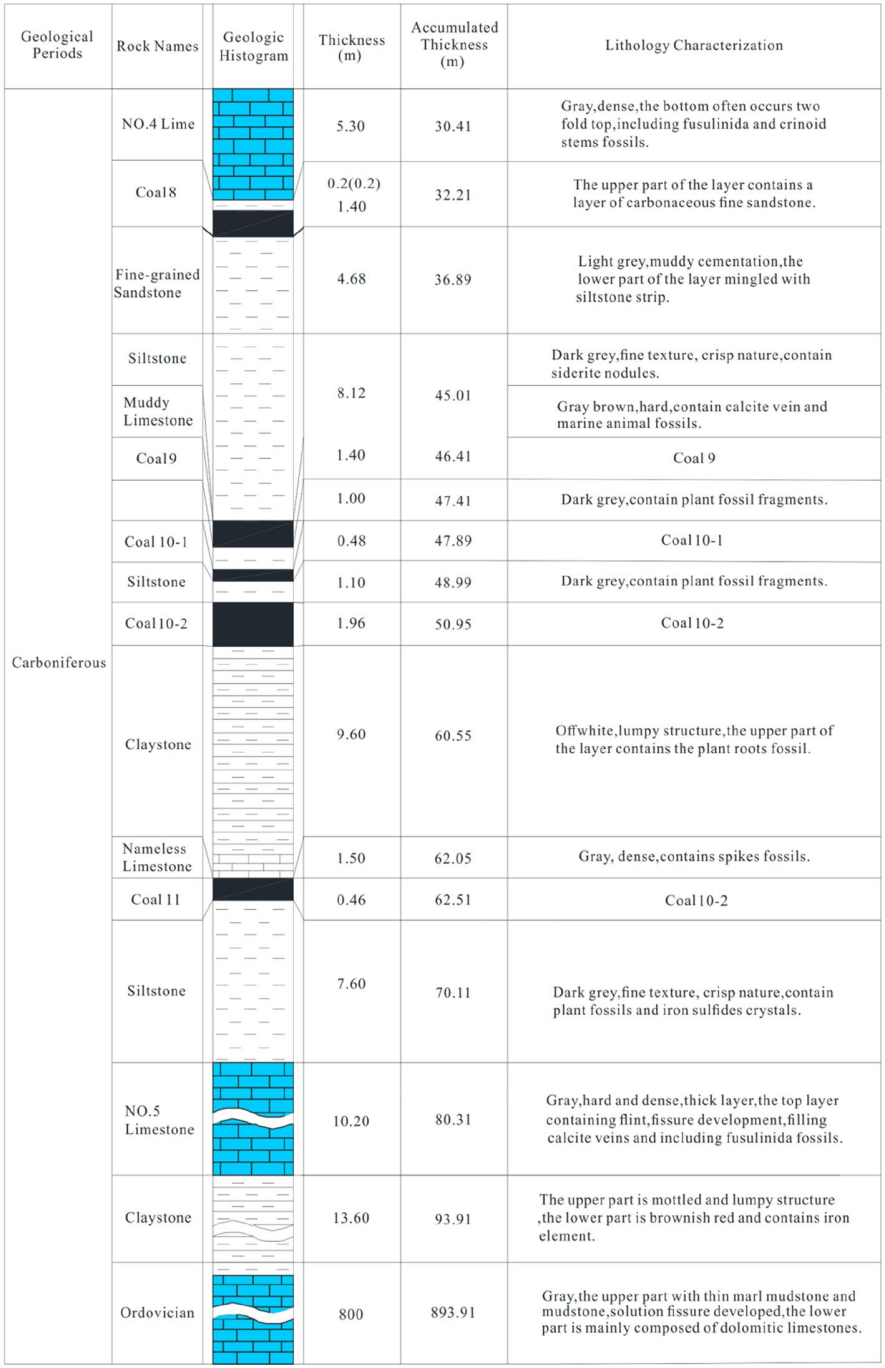
Stratigraphic column of 81006 working face in Caozhuang Coalmine.

**Figure 4:**
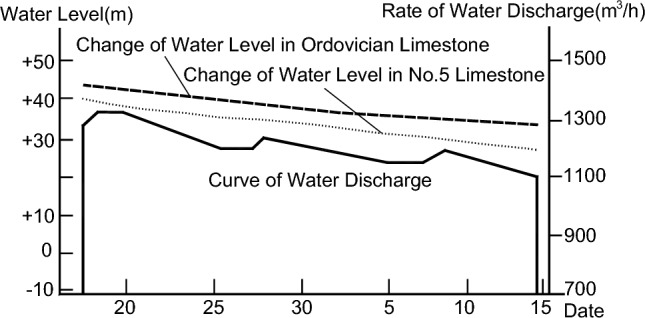
Relationship between water tables and release amount of water.

The 81006 working face in Caozhuang Coalmine has a strike length of 800 m and a tilt width of 120 m. Its elevation ranges between −358.7 and −397.7 m, with a depth between 479 and 518 m (Fig. 2). Coal seam #8, which has an average thickness of 1.96 m and a dip angle of 20°, will be mined in this working face. The direct roof of the coal seam is No. 4 limestone with a thickness of 5.3 m, while the direct floor is composed of fine sandstone with a thickness of 4.68 m. The distance between coal seam #8 and floor No. 5 limestone aquifer is 37.9 m, and the distance between No. 5 limestone aquifer and Ordovician limestone aquifer is 13.6 m. The thickness of No. 5 limestone is 10.2 m, while the thickness of the Ordovician limestone is more than 800 m (Fig. 3). Through geological and hydrogeological exploration data, it was found that there is a close relationship between No. 5 limestone aquifer and Ordovician limestone aquifer in Caozhuang Coalmine through faults. Figure 4 shows the water level change curves of No. 5 limestone aquifer and Ordovician limestone aquifer during the test of releasing groundwater engineering carried out in April 1988, which indicates that the water level change characteristics of both aquifers are the same. Therefore, No. 5 limestone aquifer and Ordovician limestone aquifer are considered as the same aquifer. This conclusion is supported by water pressure data, where the water pressures of No. 5 limestone aquifer and Ordovician limestone aquifer, obtained from hydrological observation holes in the 81006 working face, are 1.70 MPa and 1.84 MPa, respectively, and the water pressure difference is approximately equal to the distance between No. 5 limestone and Ordovician limestone.

### Evaluation of water inrush from floor in 81006 working face

According to Regulations for Mine Water Prevention and Control^24^, we use water-inrush coefficient to judge whether water inrush from floor in 81006 working face happens. That is:

1 $$ T = \frac{P}{M - C} $$

where *T* is water-inrush coefficient, MPa/m. *P* is water pressure of aquifer, MPa. *M* is the thickness of aquicludes, m. *C* is destroyed floor depth by underground pressure, m. The standard for the judge is *T* ≥ 0.06 MPa/m, water inrush, otherwise no water inrush.

According to Pillar Design and Mining Regulations under Buildings, Water, Rails and Major Roadways^25^, the destroyed floor depth by underground pressure (*C*) can be calculated with following equation:

2 $$ C = 0.0085H + 0.1665\alpha + 0.1079L - 4.3579 $$

where *H* is mining depth, m. *α* is dip angle of coal seam, °. *L* is tilt width of working face, m.

Based on Eq. (2) and Eq. (1), we calculate the water-inrush coefficient of 81006 working face. Take *H* = 518 m, *α* = 20°, *L* = 120 m into Eq. (2), get *C* = 16.32 m. Take *P* = 1.70 MPa, *M* = 37.9 m into Eq. (1), get *T* = 0.08 MPa/m. Since the water-inrush coefficient of 81006 working face is more than 0.06 MPa/m, there would be water inrush from floor in 81006 working face if we do not take water prevention and control measures.

### Backfill system constitution

#### Backfill material and backfill way

In Caozhuang Coalmine, glue material, coal gangue powder, and fly ash are used as backfill materials to create a paste-like filling in the goaf of 81006 working face. The proportion relationship of the three materials is 1.8:6:6, respectively. The fill slurry quality percentage concentration is 60% and the specific gravity is 1.56. Table 1 provides information on the intensities of the paste-like fill used in the No. 81006 working face of Caozhuang Coalmine.

**Table 1:** Paste-like fill’s intensities in No. 81006 working face of Caozhuang Coalmine.

1 day load (KN)	Average strength (MPa)	3 day load (KN)	Average strength (MPa)	7 day load (KN)	Average strength (MPa)	28 day load (KN)	Average strength (MPa)
0.88	0.19	4.5	1.12	10.4	1.83	17	3.24
0.92		5.71		7.53		15.35	
0.98		6.54		9.46			

The backfilling process in the 81006 working face of Caozhuang Coalmine uses gravity transportation. This means that the backfill slurry flows automatically through the feeding hole and pipelines to the underground working face goaf. The backfill orifice is located at an elevation of + 114.4 m, and the hole bottom elevation is − 240 m. The hole has a depth of 354.4 m, while the working face's elevation ranges from −358.7 to −397.7 m. The length of the backfill pipeline is 1850 m, and the backfill height is 512 m. The ratio between the pipe length and backfilling depth is 3.6.

#### Ground system constitution

**Figure 5:**
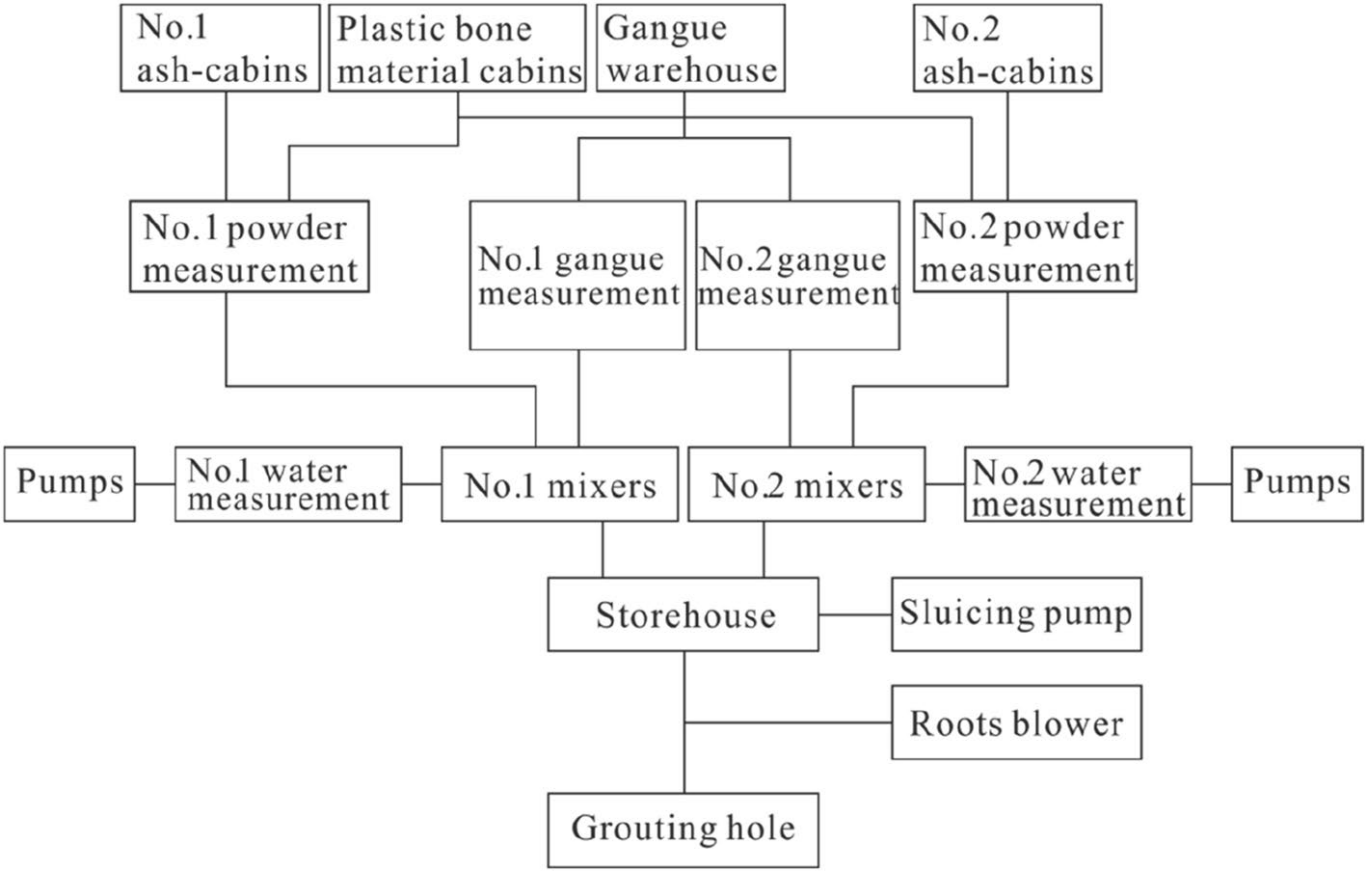
Mixing system of burdening.

1. The making material system in this project is composed of several components, including a crusher, a screening machine, a vertical hoist, 7 waste rock crushing material conveying belts, and a forklift truck. The crusher, screening machine, and vertical hoist have a capacity range of 100–150 t/h. The finished size of the waste rock is less than 5 mm, and the overall backfill capacity of the system is estimated to be 100–120 t/h. This system is designed to efficiently process and transport the waste rock for use in the backfilling process of the working face.
2. The storage material system includes two fly ash silos with a volume of 200 m^3^, one glue material container with a volume of 100 m^3^, one coal gangue powder tank with a volume of 150 m^3^, one material storage shed with a volume of 3000 m^3^, four conveyor belts, and one loading forklift. Please refer to Figs. 5 and 6 for more information.

**Figure 6:**
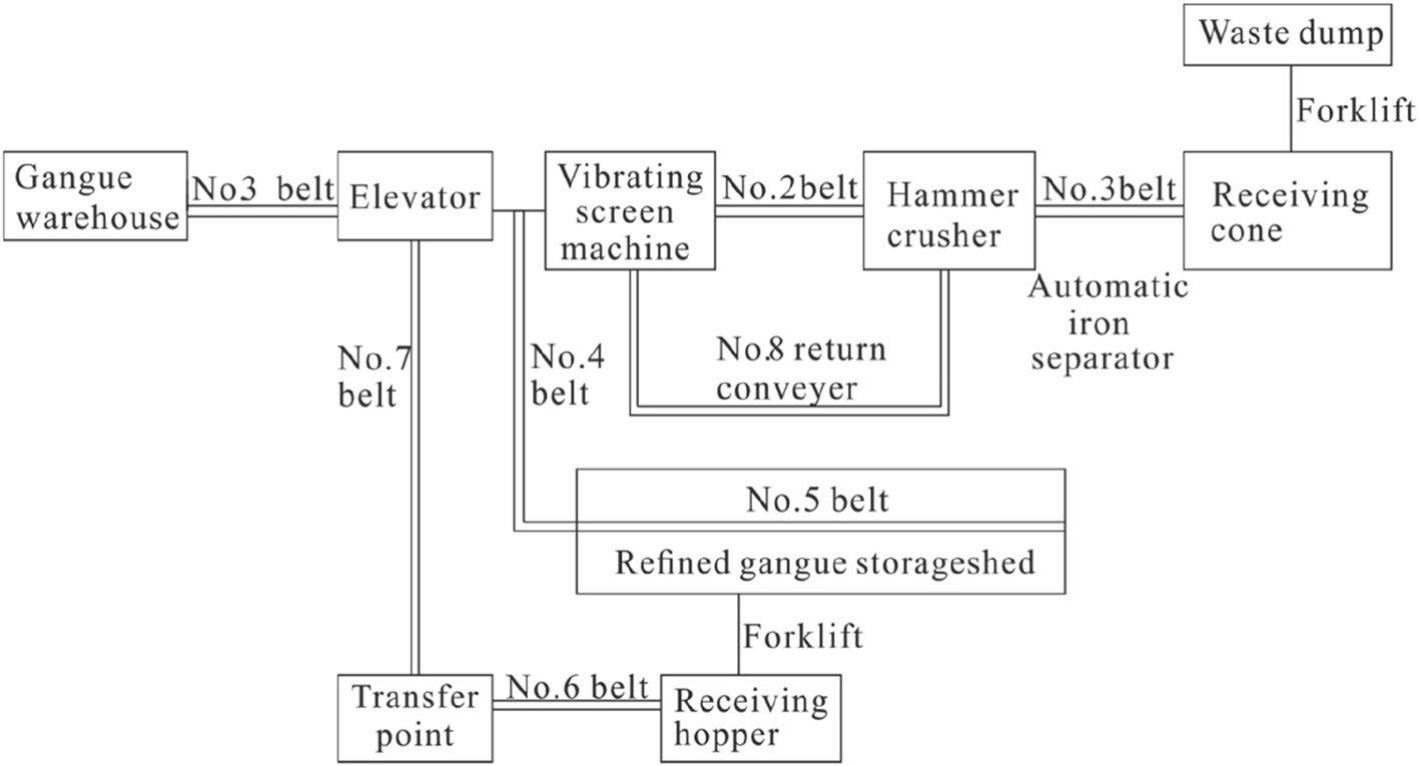
Flow chart of crushing and screening.
3. The power and water supply system of the backfilling operation features a dedicated substation that includes two transformers with a capacity of 800 KVA and associated equipment. The incoming line voltage is 6 kV, while the output line voltages are 380 V and 220 V. The total equipment load is 665 KW, with the crushing system accounting for 440 KW, the pulping system for 225 KW, and the control and lighting system for 25 KW. The water supply comprises a 500 m^3^ reservoir, five pumps, five pipelines, and one measuring control device. The backfill water source is underground sewage, which provides a water supply capacity of 200 m^3^/h.
4. The automatic control and measurement system comprises a master device, a centralized control device, 28 automatic control points, a pneumatic control device, 2 blenders, 4 screw conveyors, and 4 measuring control equipment.
5. The monitoring communication system comprises a TV monitor system, 16 cameras, two ground fixed telephones, five underground fixed telephones, and eight interphones.
6. The emergency disposal system comprises an emergency water tank, a rinse water pump, and a pressure fan.

#### Underground system constitution

**Figure 7:**
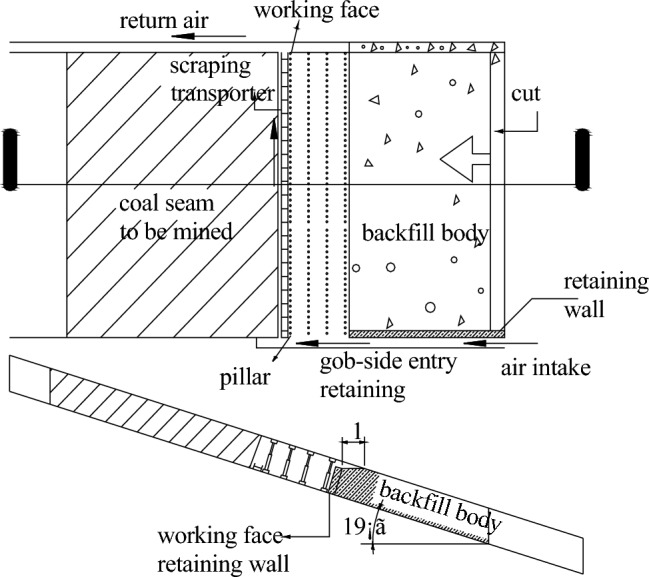
Backfill mining layout plans of working face.

1. The pipe conveyor system comprises a ground vertical borehole and an underground pipeline. A composite wear-resistant ceramic pipe, with a bore diameter of Φ146 × 13 mm and a length of 350 m, is inserted into the ground vertical borehole. The underground pipeline is composed of composite wear-resistant ceramic pipes with a bore diameter of Φ108 × 6 mm and a length of 1850 m. The pipeline is equipped with pressure gauges for monitoring.
2. The block slurry system in the working face is comprised of a block slurry drape and a block slurry wall along the roadway. The block slurry drape is made up of filter bags, while the block slurry wall is composed of block slurry cloths, steel plates, and dense pillars. In order to prevent the outflow of backfill slurry, the block slurry wall must be securely fixed and tightly connected to both the roof and the floor (as shown in Fig. 7).

#### Roadway layout

To prevent backfill slurry outflow and reduce pressure on the gob side entry retaining, the roadway layout follows the coal seam tendency and up-dip mining is used as the advance mining method. The 81006 working face is divided into three inclined short wall working faces, namely 81006I, 81006II, and 81006III, to be mined and filled sequentially. 81006I has one cut and one air return way. Transport lane tunneling is not required before working face mining in 81006I, but during working face mining. Gob side entry retaining is utilized as the air return way for 81006II. A transport lane is situated in the middle of the 81006II and 81006III double-unit face. The ventilation mode used is "Z" type ventilation (Fig. 2).

#### Coal mining method and technology

The mining methods employed in the project include the inclined short wall advancing mining method and high-grade general mining method. The shearer used is the DW-150 type, the scraper conveyor is the SGB-150C type, and the roof support consists of a single hydraulic prop with support that reverts to four ways back to one. The management of the roof is through the gangue slurry filling method. The coal seam is mined using the full-seam mining technique, with a cycle progression of 1 m. The method of continuous mining and interval filling is adopted, where each working face is filled with 4 m of material once it has advanced 4 m.

The technological process of the mining operation includes drilling and charging, blasting, coal cutting, using a blasting pressure gun, loading coal with a loose machine, moving the scraper conveyor, building basic pillars, pulling the roof supports, fixing the grouting curtain blocks, withdrawing the last row of pillars, constructing the slurry retaining wall, backfilling, and solidifying the backfill material. To control the roof, the working face uses three or four rows of pillars, with a maximum control roof distance of 4.4 m and a minimum control roof distance of 3.4 m.

#### Backfill technology

The mining process of the working face progresses every 4 m, followed by the preparation and implementation of the gangue and slurry backfill. The backfilling process consists of several steps, including the preparation of the backfill, water filling for pigging, grouting the slurry, filling the gangue, additional grouting of the slurry, water injection, water filling for pigging, and finally, blowing the pipe with high-pressure wind.

#### Mechanical parameter testing of filling body

**Figure 8:**
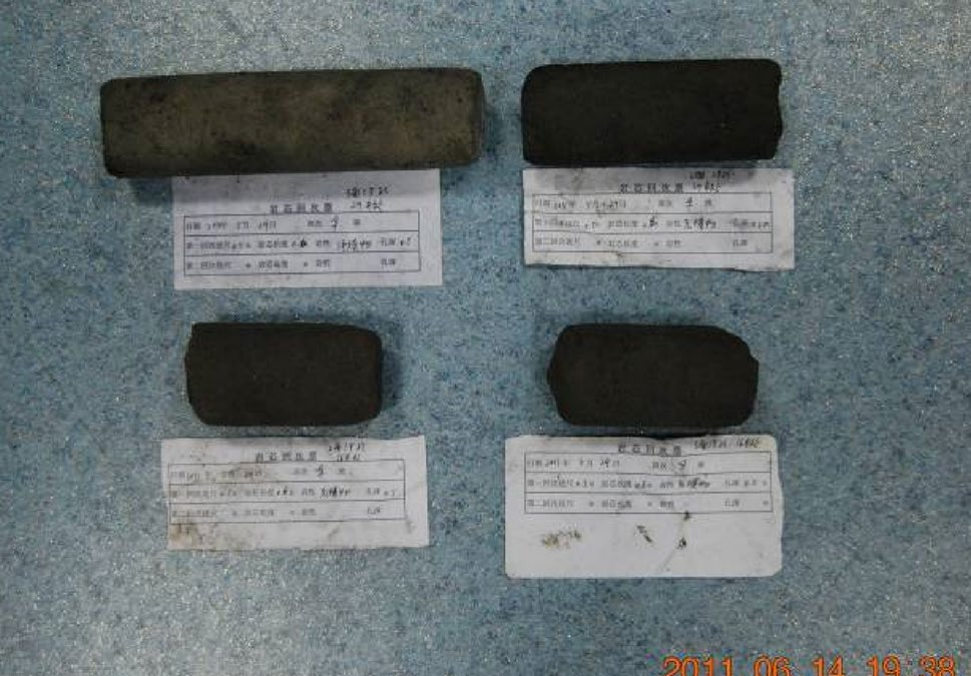
The rock cores of filling material collected on site.

The on-site sampling of rock cores is shown in Fig. 8. The upper left core was taken to 29 m from hole 2 on surface III, the upper right core was taken to 29 m from hole 2 on surface II, the lower left core was taken to 16 m from hole 1 on surface II, and the lower right core was taken to 16 m from hole 1 on surface III.

**Figure 9:**
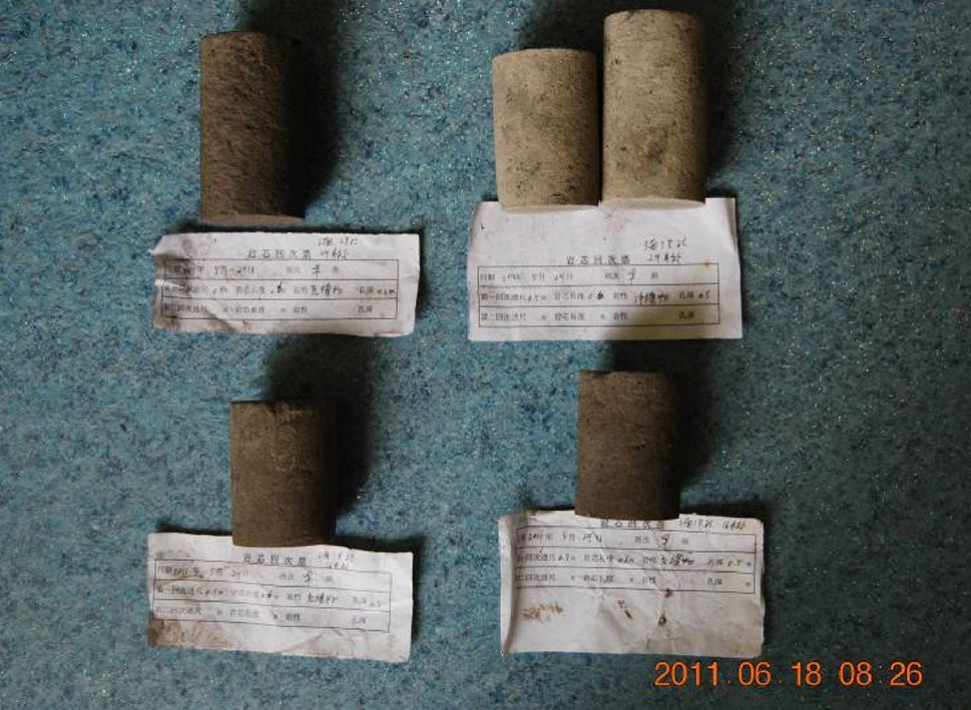
The standard rock cores of the filling material.

A cylindrical rock core is used as the standard sample, with a diameter of 50 mm, a permissible variation range of 48–54 mm, a height of 100 mm, and a permissible variation range of 95–105 mm. The standard rock cores of the filling material are shown in Fig. 9. Among them, the lower left is at 16 m from hole 1 on surface II, with specimen number A-1. The upper left is at 29 m from hole 2 on surface II, with specimen number B-1. The lower right is at 16 m from hole 1 on surface III, with specimen number C-1. The upper right is at 29 m from hole 2 on surface III, with specimen numbers D-1 and D-2.

1. Calculation of the uniaxial compressive strength
  The uniaxial compressive strength is computed by the following formula.
  $$ \sigma_{c} = \frac{P}{A} $$
  where *σ*_*c*_ is the uniaxial compressive strength, MPa; *P* is the failure load, N; *A* is the cross-sectional area, mm^2^. The results are shown in Fig. 10.

**Figure 10:**
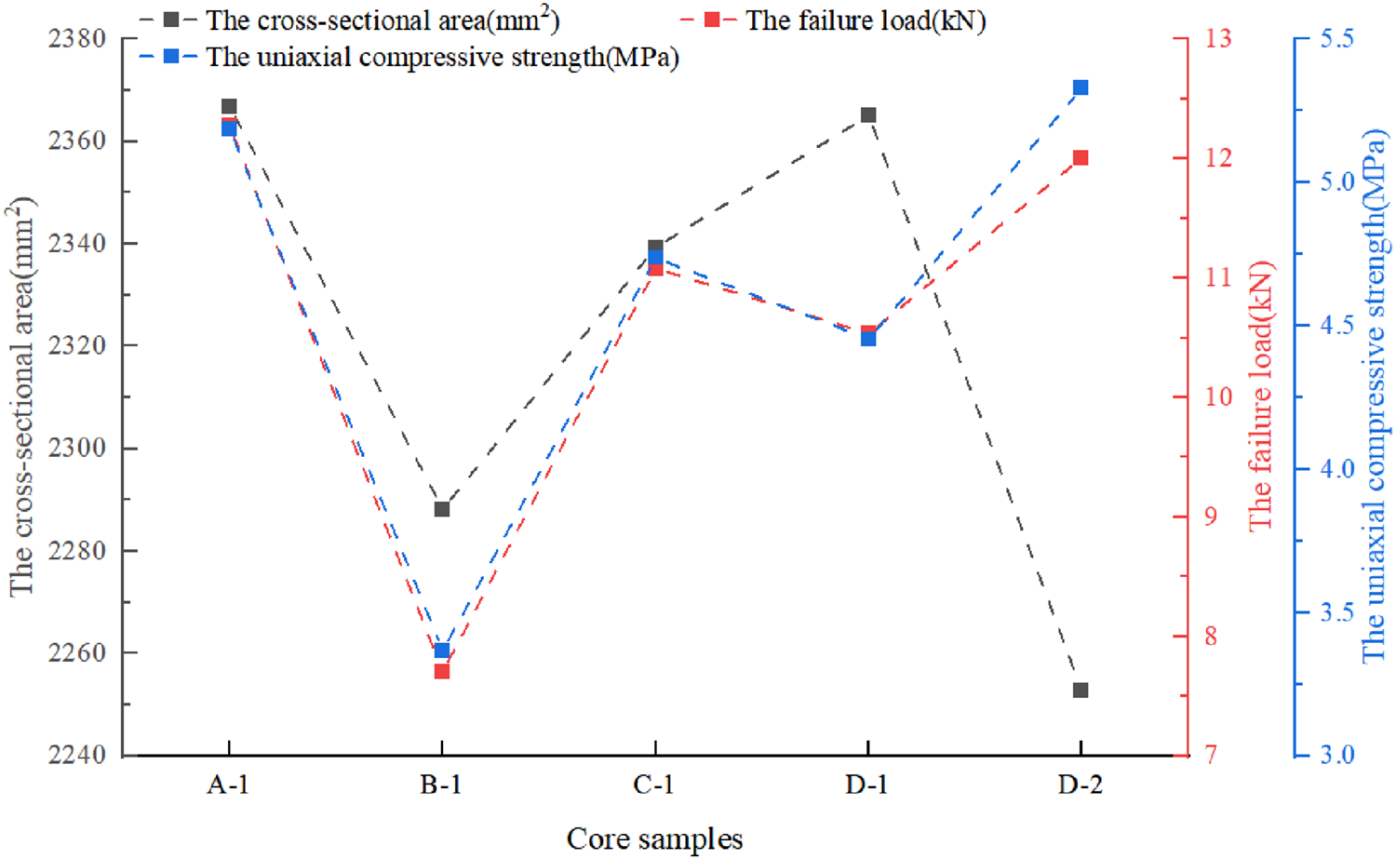
The results of the uniaxial compressive strength for A-1, B-1, C-1, D-1, D-2.
2. Calculation of the elastic modulus
  The average elastic modulus is computed by the following formula.
  $$ E_{av} = \frac{{\sigma_{b} - \sigma_{a} }}{{\varepsilon_{b} - \varepsilon_{a} }} $$
  where *E*_*av*_ is the average elastic modulus, MPa; *σ*_*b*_ is the stress value at the endpoint of the straight line segment on the relationship curve between the stress and the strain, MPa; *σ*_*a*_ is the stress value at the starting point of the straight line segment on the relationship curve between the stress and the strain, MPa; *ε*_*b*_ is the longitudinal strain of the stress *σ*_*b*_; *ε*_*a*_ is the longitudinal strain of the stress *σ*_*a*_. The relationship curves between the stress and the strain are shown in Fig. 11, and the results of the elastic moduli for A-1, B-1, C-1, D-1, D-2 are shown in Fig. 12.

**Figure 11:**
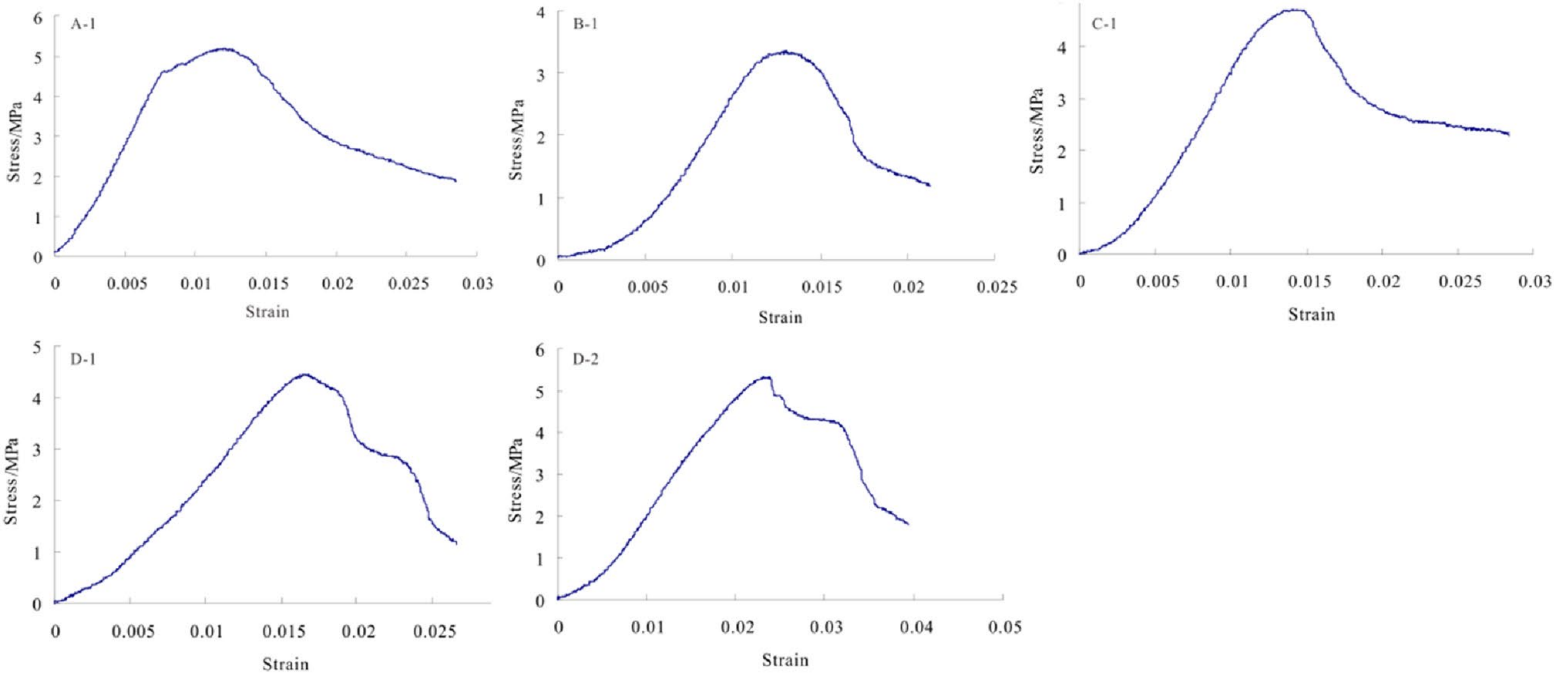
The relationship curves between the stress and the strain for A-1, B-1, C-1, D-1, D-2.

**Figure 12:**
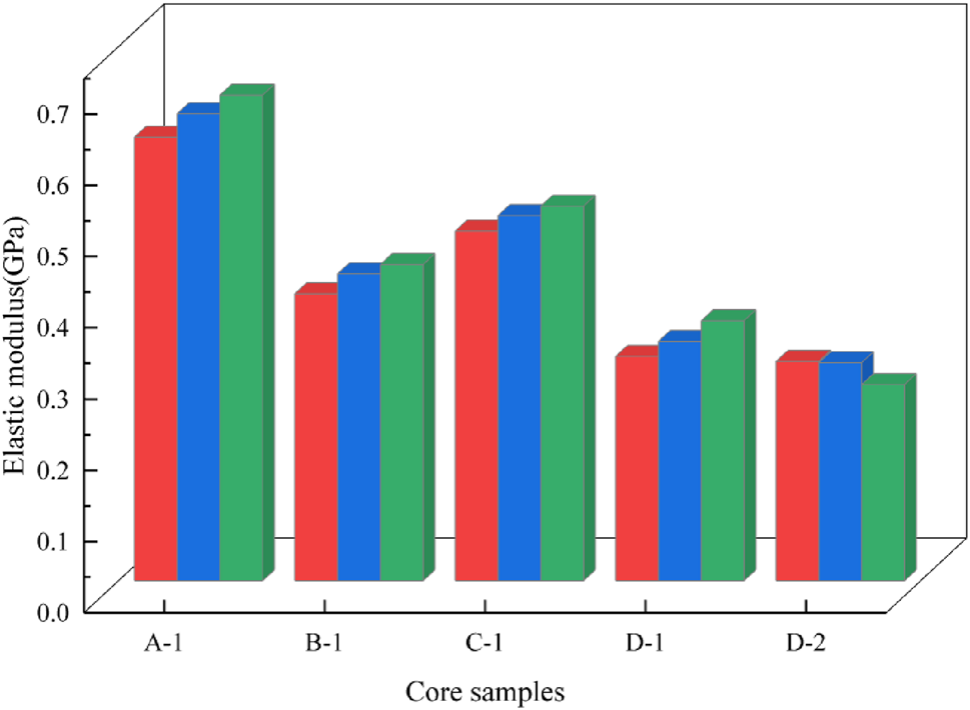
The results of the elastic moduli for A-1, B-1, C-1, D-1, D-2.

It can be seen that there is not much difference in the density of the rock samples. The failure load and the uniaxial compressive strength of B-1 are significantly lower than other specimens. The elastic moduli of D-1 and D-2 are not significantly different due to being taken from the same rock core, but they are relatively low compared to other specimens. Due to size effects, the Poisson's ratios of D-1 and D-2 have a certain degree of dispersion. Considering that D-1 is close to the processing requirements of standard specimens, the Poisson's ratio of D-1 is used as the standard. The unidirectional compressive strength of the filling body in the 81006 working face is about 4.5 Mpa, and the elastic modulus *E* is 0.34 Gpa. The unidirectional compressive strength is greater than the designed strength value (3.0 Mpa), meeting the requirements.

### The destroyed floor depth detection of 81006 working face

#### Detection method of destroyed floor depth

**Figure 13:**
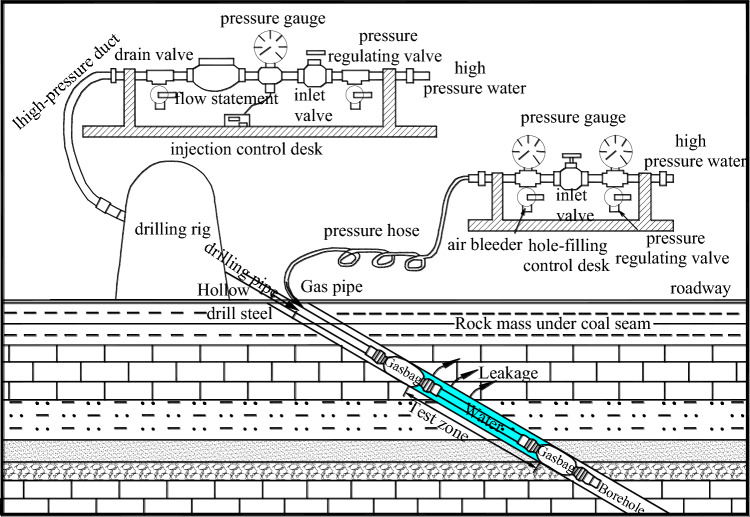
Double side seal borehole water injection device.

We employ the downhole drilling section water injection method to determine the depth of destroyed floor in a working face, utilizing the double side seal borehole water injection device. This device is comprised of a control unit and instrumentation system located externally to the drilling operation, and a double-sided seal borehole water injection testing tube situated internally within the drilling mechanism (Fig. 13).

The depth of destroyed coal seam floor is measured through section water injection in boreholes using the double side seal borehole water injection device. The process involves selecting suitable observation positions around the underground working face, such as mining roadways near the stopping line of the working face, and making oblique drilling towards the direction of the goaf floor. The amount of water loss is then measured in pieces using the double side seal borehole water injection device. The depth of destroyed coal seam floor is determined based on the leakage observed in the drill after coal mining.

Two connecting capsules are installed on both ends of the test tube, which remain in a state of static contraction and allow the test tube to be moved by the drill pipe into the borehole anywhere. The capsules can be expanded by injecting air from an underground high-pressure source through an air pressure regulating valve, an instrument, and a pressure hose to press against the borehole wall, forming a double-sided plugging borehole section with a length of 1 m. Stable pressure water from an underground high-pressure water source is injected into the double-sided plugging borehole section through the drill pipe, regulating valve, and pressure flow meter. The water flow through fractures in the wall of the double-sided plugging borehole section can be measured by a water flow meter. According to hydrodynamic theory, when the water injection pressure is stable, the water injection flow rate depends on the rock permeability and the size of the fracture, i.e., water injection flow increases as the rock permeability coefficient and the degree of fracture development increase. The measured results indicate that, under the condition of a stable water injection pressure of 0.1 MPa, if the coal floor strata are not destroyed by underground pressure, the water injection flow rate value for each 1 m borehole section is less than 1 L/min, and may even tend to zero. Conversely, the water injection flow rate value is greater than 1 L/min in destroyed floor strata.

#### The scene detection

To determine the destroyed floor depth of the 81006 paste-like backfill working face, we conducted four drillings named *d*_1_, *d*_2_, *d*_3_, and *d*_4_ from the track roadway towards the goaf floor. *d*_1_ and *d*_2_ were at the same site, while *d*_3_ and *d*_4_ were at another site. The double side seal borehole water injection device was used to measure the destroyed depth of the coal seam floor. Table 2 shows the design parameters of the four drills, while Table 3 shows the measurement parameters of the drills.

**Table 2:** Parameters of four drills.

Drills	*d*_1_	*d*_2_	*d*_3_	*d*_4_
Azimuth angle (°)	240	240	321	321
Dip angle (°)	3	−3	−1	−8
Length (m)	91.8	87	91.8	77

**Table 3:** Injected water loss amount of *d*_1_, *d*_2_, *d*_3_ and *d*_4_.

Length (m)	*d*_1_ (L/min)	*d*_2_ (L/min)	*d*_3_ (L/min)	*d*_4_ (L/min)
1	2.0	2.4	2.0	2.0
2	2.0	2.4	2.0	2.0
3	2.0	2.4	2.0	2.0
4	2.0	2.4	2.0	2.0
5	2.0	2.4	2.0	2.0
6	2.0	2.4	2.0	2.0
7	2.0	2.4	2.0	2.0
8	2.0	2.4	2.0	1.8
9	2.0	2.4	2.0	1.8
10	2.0	2.4	2.0	1.8
11	2.0	2.4	2.0	1.8
12	2.0	2.4	2.0	1.8
13	2.0	2.4	2.0	2.0
14	1.9	2.4	2.0	2.0
15	1.9	2.4	2.0	0
16	1.9	2.4	2.8	0
17	1.8	2.4	3.0	0
18	1.8	2.4	6.0	0
19	1.8	2.0	6.0	0
20	1.8	2.0	0	0
21	1.6	2.0	0	0
22	1.6	2.0	0	0
23	1.6	1.6	0	0
24	1.6	1.6	0	0
25	1.6	1.6	0	0
26	1.6	0	0	0
27–42	0	0	0	0

#### Detection results analysis

**Figure 14:**
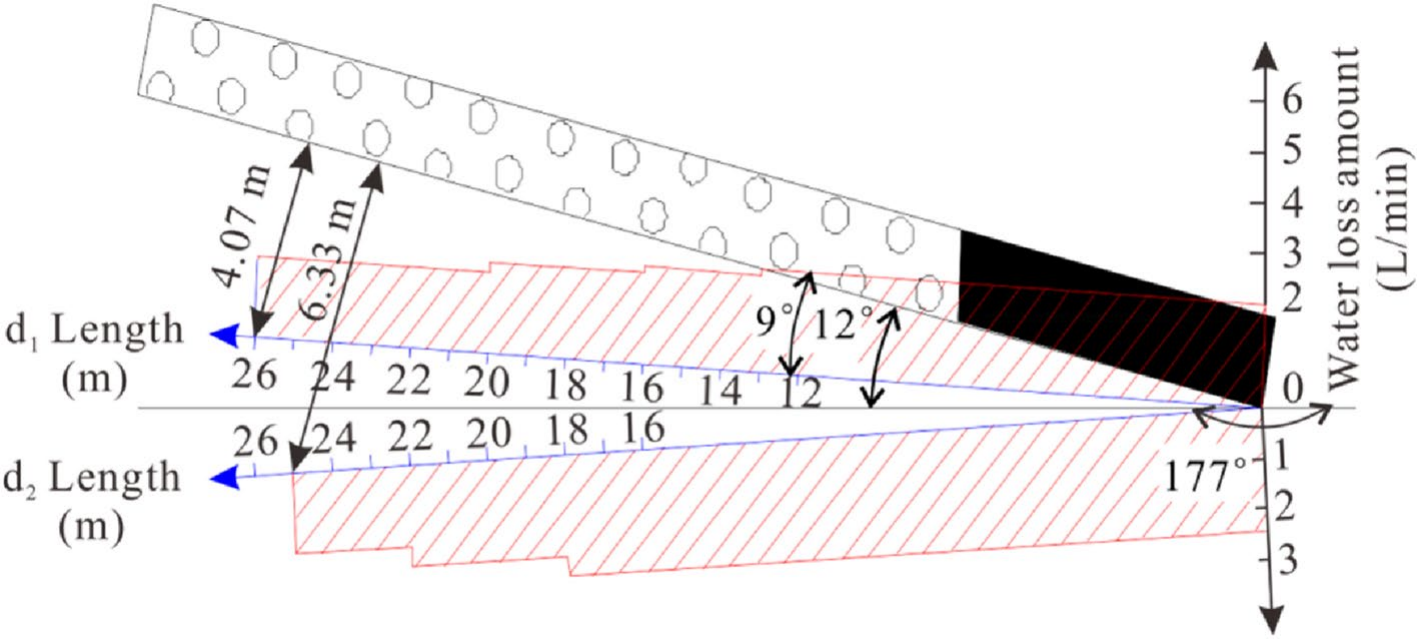
Leakage of *d*_1_ and *d*_2_ drills in the 2nd drilling field of No. 81006 working face.

**Figure 15:**
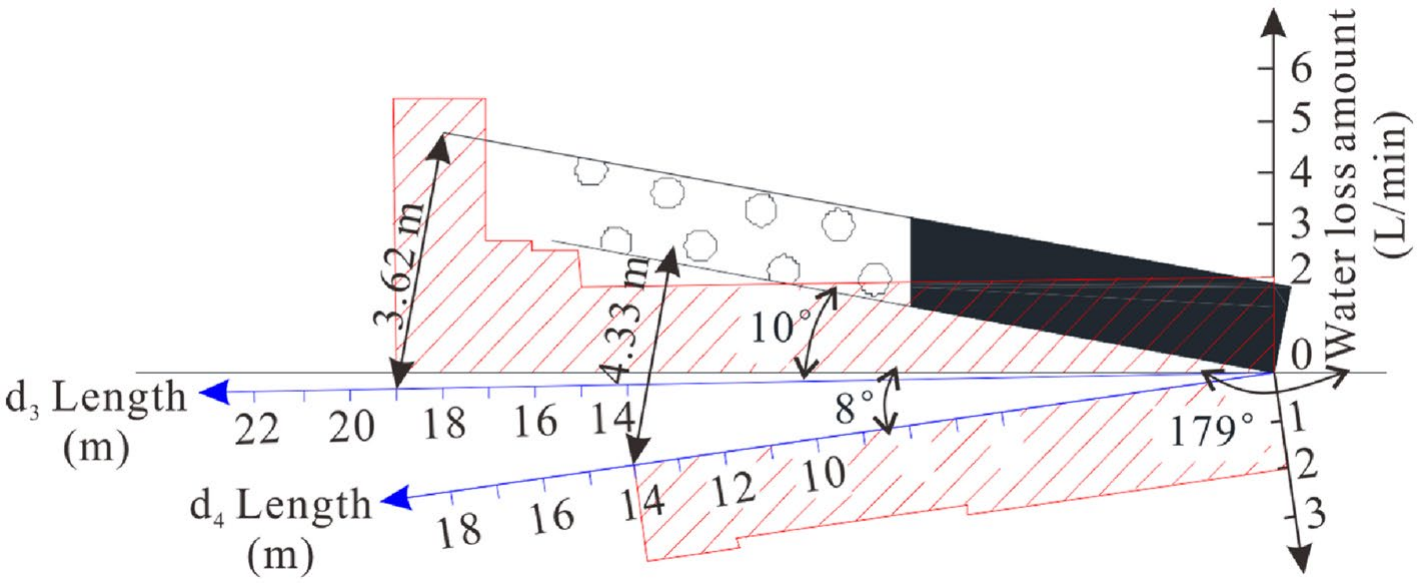
Leakage of *d*_3_ and *d*_4_ drills in the 1st drilling field of No. 81006 working face.

Based on the data in Table 3, we have plotted Figs. 14 and 15 to illustrate the injected water loss amount of *d*_1_ and *d*_2_, and *d*_3_ and *d*_4_, respectively. As shown in the figures, the destroyed floor depths for *d*_1_, *d*_2_, *d*_3_, and *d*_4_ are 4.07 m, 6.33 m, 3.62 m, and 4.33 m, respectively. Thus, we can conclude that the maximum destroyed floor depth in the 81006 paste-like backfill working face is 6.33 m.

Based on Eq. (1), we can calculate the water-inrush coefficient of the 81006 paste-like backfill working face. By substituting *P* = 1.70 MPa, *M* = 37.9 m, and *C* = 6.33 m into Eq. (1), we obtain *T* = 0.05 MPa/m. Since the calculated water-inrush coefficient is less than 0.06 MPa/m, it indicates that water inrush from the floor in the 81006 paste-like backfill working face is unlikely to occur.

### Mechanism analysis of reducing the destroyed floor depth of paste-like backfill working face

**Figure 16:**
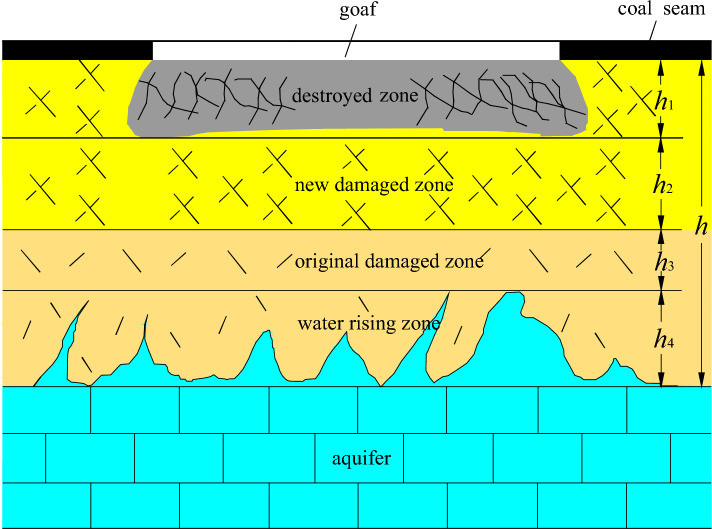
The model of lower four-zone theory.

Based on the "low four-zone theory" in China^26^, the strata in the goaf area between the mined coal seam and aquifer can be categorized into four zones: the destroyed zone caused by underground pressure, the new damaged zone, the original damaged zone, and the original confined water rising zone (Fig. 16). The destroyed zone caused by underground pressure refers to the floor strata in which the rocks are severely damaged by underground pressure, resulting in a complete loss of water-resisting ability. The new damaged zone refers to the floor strata where rocks are damaged by underground pressure but still maintain their water-resisting ability. The original damaged zone refers to the floor strata where rocks are undamaged by underground pressure and have original water-resisting ability. The original confined water rising zone refers to the floor strata where the confined water in the aquifer rises along the original rock fracture. According to the theory of damage fracture mechanics, the floor strata between the coal seam and aquifer are in the original damaged state before coal seam mining. However, the stress concentration occurs after coal seam mining and leads to fractures in the original damaged zone, which extends to form the new damaged zone. As the working face advances, the underground pressure in the floor also increases, causing an additional increase in the thickness of the damage zone and a gradual decrease in the thickness of the original damage zone. When the fractures in the new damaged zone extend to crisscross and connect with each other, the destroyed zone caused by underground pressure begins to form. As the working face continues to advance and the underground pressure further increases, both the new damaged zone and destroyed zone caused by underground pressure gradually increase while the original confined water rising zone gradually decreases.

According to “low four-zone theory”, the condition of water-inrush from floor is as follows:

1. If *h*_*3*_ ≠ 0, then no water-inrush;
2. If *h*_*3*_ = 0, *h*_*2*_ ≠ 0, and the aquifer pressure is less than compressive strength of new damaged zone, then no water-inrush;
3. If *h*_*3*_ = 0, *h*_*2*_ ≠ 0, and the aquifer pressure is more than compressive strength of new damaged zone, then water-inrush;
4. If *h*_*3*_ = 0, *h*_*2*_ = 0, then water-inrush.

**Figure 17:**
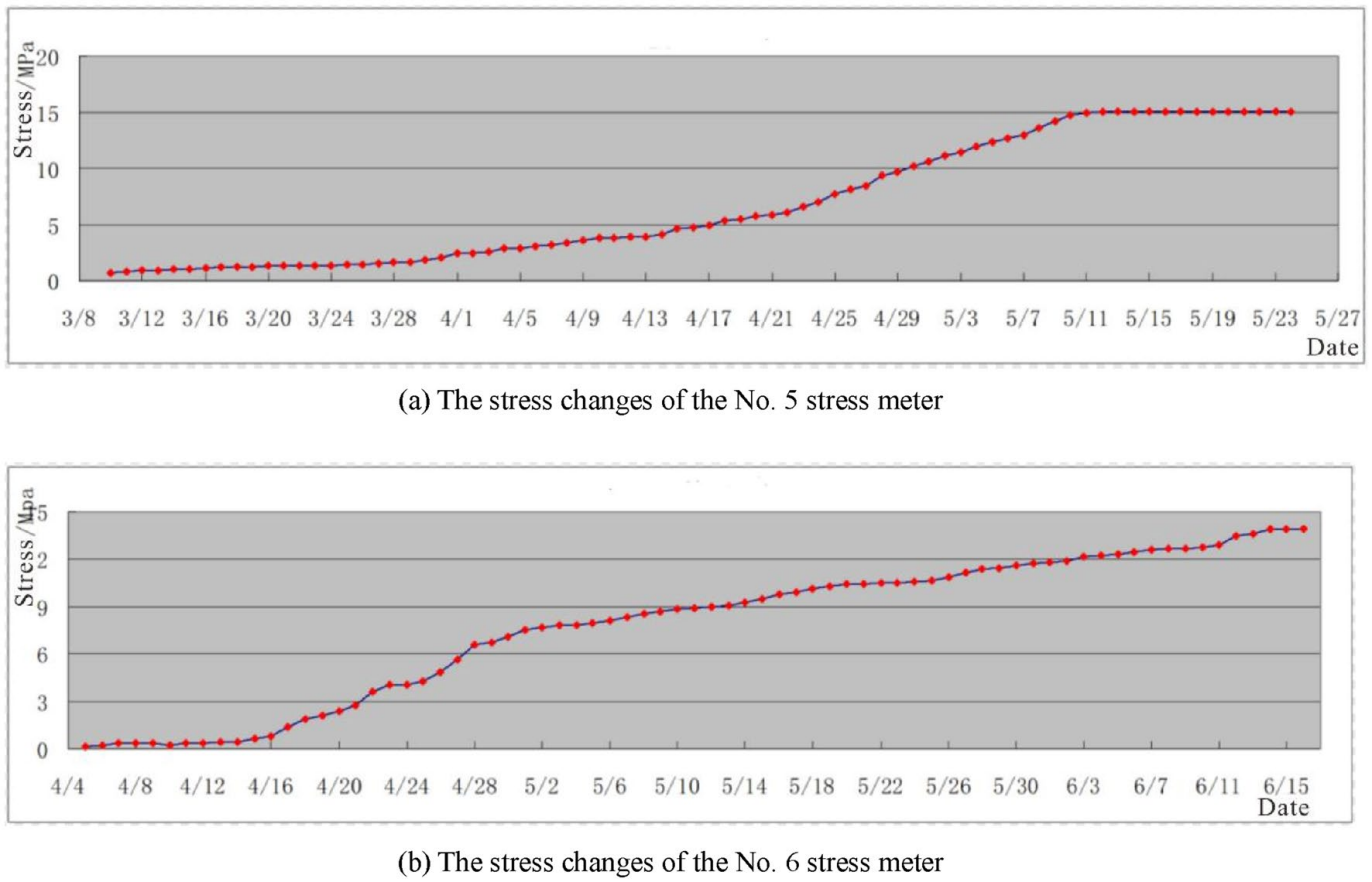
The stress changes of paste-like backfill mining.

Preventing water-inrush from the floor is a crucial technology in coal mining, requiring the inhibition of the transformation of the original damaged zone into a new damaged one and the control of the evolution of the latter into a destroyed zone caused by underground pressure^27^. An effective approach to achieve this goal is the use of paste-like backfill in the working face, which can significantly reduce the concentration of underground pressure in the filled goaf. Measured data of stress changes in the filling body (Fig. 17) confirm that about two months after the implementation of the paste-like backfill, stress meters 5# and 6# reached a stable state with stress values of 15.06 MPa and 13.92 MPa, respectively. These values are close to the original stress of 13.75 MPa, indicating that the paste-like backfill can effectively support the weight of the overlying strata.

## Conclusions

1. The use of paste-like backfill mining technology significantly reduces the depth of damage to the working face floor caused by mining pressure. According to the exploration data of the 81006 working face, when the No. 8 coal seam is mined by using the paste-like backfill technology, the maximum depth of floor failure is 6.33 m; When mined by using the method of roof caving for the 8812 working face, the destroyed floor depth is 36.5 m. The depth of damage to the working face floor through the paste-like backfill technology is only 17% of that through the roof caving.
2. The paste-like backfill technology is an effective measure to prevent water inrush from floor. When the No. 8 coal seam is mined by using the roof caving, the floor strata are seriously damaged by the mining pressure, causing the expansion of original fractures and the generation of a large number of new fractures, which connect with each other and form a fracture network, resulting in significant changes of aquifers in the water content and grouting amount. The use of paste-like backfill technology significantly reduces the damage of mining pressure to the floor strata, and can effectively prevent the water inrush from floor.
3. The paste-like backfill filling method can efficiently control the mining-induced pressure and inhibit or reduce the conversion among the original damaged zone, new damaged zone, and destroyed zone caused by underground pressure.

## Data Availability

The data that support the findings of this study are available from the corresponding author upon reasonable request.
